# Development and validation of a postoperative delirium prediction model for pediatric patients: A prospective, observational, single-center study

**DOI:** 10.1097/MD.0000000000025894

**Published:** 2021-05-21

**Authors:** Nan Lin, Kexian Liu, Jingyi Feng, Ruan Chen, Yan Ying, Danni Lv, Yue Zhou, Hongzhen Xu

**Affiliations:** a Nursing Department; b Otorhinolaryngology Head and Neck Surgery; c General Endoscopic Surgery; d Surgical Oncology, the Children's Hospital, Zhejiang University School of Medicine, National Clinical Research Center for Child Health, China.

**Keywords:** area under the receiver operating characteristics curve, pediatric, postoperative delirium, predictors, prevalence, risk factors

## Abstract

Postoperative delirium is a serious complication that relates to poor outcomes. A risk prediction model could help the staff screen for children at high risk for postoperative delirium. Our study aimed to establish a postoperative delirium prediction model for pediatric patients and to verify the sensitivity and specificity of this model.

Data were collected from a total of 1134 children (0–16yr) after major elective surgery between February 2020 to June 2020. Demographic and clinical data were collected to explore the risk factors. Multivariate logistic regression analysis was used to develop the model, and we assessed the predictive ability of the model by using the area under the receiver operating characteristics curve (AUROC). Further data were collected from another 100 patients in October 2020 to validate the model.

Prevalence of postoperative delirium in this sample was 11.1%. The model consisted of 5 predictors, namely, age, developmental delay, type of surgery, pain, and dexmedetomidine. The AUROC was 0.889 (*P* < .001, 95% confidence interval (CI):0.857–0.921), with sensitivity and specificity of 0.754 and 0.867, and the Youden of 0.621. The model verification results showed the sensitivity of 0.667, the specificity of 0.955.

Children undergoing surgery are at risk for developing delirium during the postoperative period, young age, developmental delay, otorhinolaryngology surgery, pain, and exposure to dexmedetomidine were associated with increased odds of delirium. Our study established a postoperative delirium prediction model for pediatric patients, which may be a base for development of strategies to prevent and treat postoperative delirium in children.

## 1 Introduction

Delirium is a frequent, underrecognized, complex neurologic dysfunction in the setting of serious illness.^[1,2]^ According to the American Psychiatric Association's Diagnostic and Statistical Manual of Mental Disorders, Fifth Edition, characteristics of delirium include acute onset, fluctuating course, and disturbances of cognitive abilities that do not occur within the context of a severely reduced level of arousal.”^[3]^ Delirium is an acute non-traumatic brain injury that relates to longer hospital length of stay (LOS), increased morbidity and mortality, poor long-term outcomes, increased hospital costs, and higher demand of care.^[4–6]^

In the adult population, published risk factors for postoperative delirium development include infections, drug use, length of surgery and sedation, which are very common in pediatric patients undergoing major elective surgery.^[7–9]^ An extensive research exists describing the incidence, risk factors, and outcomes of postoperative delirium in adults.^[4]^ The reported prevalence of postoperative delirium ranging from 20% to 30%, and the occurrence of postoperative delirium is associated with higher rate of postoperative stroke and increased perioperative mortality, which complicates the postoperative course in up to 56% of the cases.^[10–11]^ Although postoperative delirium in adults is widely recognized, much less is known in pediatric patients. An emerging body of pediatric research indicates that pediatric delirium is associated with young age, severity of illness, mechanical ventilation, and sedation.^[12–13]^ However, most studies focused on critically-ill children, there is little research in children after surgery. Hence the absence of widespread screening and lack of evidence-based data, postoperative delirium in pediatric patients are not yet well-described in China.

More recent research demonstrates that delirium is often reversible with early detection and treatment.^[14–15]^ Therefore, it is important to identify related risk factors before surgery and make a dedicated perioperative care path. The Cornell assessment of pediatric delirium (CAPD) is a validated screening tool designed for children of all ages and developmental abilities. We choose CAPD as the bedside screening tool to ease and expedite the evaluation of postoperative delirium.

In this study, we describe a cohort of children undergoing major elective surgery over 5 months. Our objective was to establish a postoperative delirium prediction model on the basis of the risk factors associated with pediatric patients and validate its discrimination for pediatric patients with a high risk of postoperative delirium in this study.

## 2 Materials and methods

### 2.1 Design and participants

A prospective, observational, single-center study was designed to select patients in the Children's Hospital, Zhejiang University School of Medicine, Hangzhou, China, between February 2020 to June 2020. Inclusion criteria were as follows:

1. patients younger than the age of 18 years;
2. patients after major elective surgery.

Exclusion criteria were as follows:

1. patients had delirium before the surgery;
2. patients who were admitted to the intensive care unit after surgery;
3. the doctor disagrees with the patient's participation in this study;
4. patients were participating in other projects that may affect this study.

**Figure 1:**
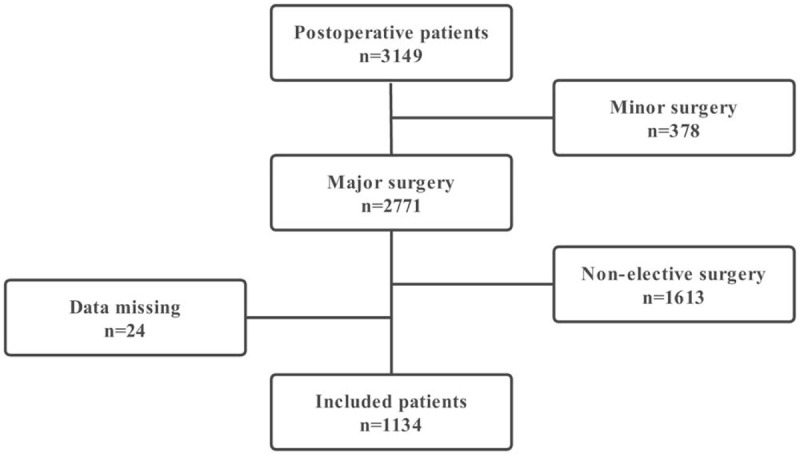
The flow chart shows the screening process of the participants.

The institutional review board of our medical center approved this observational study with a waiver of requirement for informed consent (2020-IRB-001). One thousand one hundred thirty-four patients were enrolled after major elective surgery, regardless of age or pre-existing developmental delay (Fig. 1).

### 2.2 Delirium assessment

CAPD is the only tool that has been validated across the entire pediatric age range, and it can successfully discriminate between delirium and other causes of altered mental status.^[16]^ Therefore, the authorized Chinese version of the CAPD was used as the postoperative delirium screening tool for this study.^[17]^ The CAPD consists of 8 items, scored on a Likert scale. A CAPD score of 9 or higher was considered a positive delirium screen. Developmentally delayed children were classified as delirious if they had a CAPD score of 9 or higher and the physicians confirmed alteration from the child's baseline mental status.

### 2.3 Data collection

The data was collected by 7 nurses who were trained and proficient in the CAPD assessment method after the patients recovering from general anesthesia, and the intraclass correlation coefficient among the 7 nurses was 0.825 (95%CI:0.773–0.869, *P* < .001). If the patient develops delirium, record the duration of the delirium and stop the assessment. If there is no delirium, the assessment will be performed at 9:00am and 7:00pm each day until the patient is discharged.^[18]^ Demographic and clinical data were collected upon enrollment, including age, gender, developmental status (developmental delay was defined by clinical assessment and/or parental report of developmental problems that affected the child's behavior or ability to communicate)^[16,19]^, pre-existing medical conditions, American Society of Anesthesiologists classification, and type of surgery. Putative risk factors for delirium development were extrapolated from a review of the pediatric and adult literature in medical and surgical patients.^[19]^ Preoperative data collected included: sleep duration, fluid fasting time, severity of parental anxiety using Hamilton Anxiety Scale.^[20]^ Intraoperative data collected included: length of anesthesia, length of surgery, and blood loss. Postoperative data collected included: CAPD scores,^[16]^ pain scores (Face, Legs, Activity, Cry, Consolability behavioral tool),^[21]^ body temperature and exposure to medications by categories (including narcotics, benzodiazepines, corticosteroids, anticholinergics, opioid receptor agonist, and neuromuscular blocker).

### 2.4 Statistical methods

Variables were summarized with counts and percentages, or median and interquartile range (IQR). Delirium prevalence is based on the subjects that were delirious, or delirium-free. Chi-Squared/Fisher Exact tests were performed as applicable to compare categorical factors between the delirious group and the delirium-free group. Wilcoxon rank-sum tests were performed as applicable to compare continuous factors. Multivariate logistic regression was used to assess multivariate associations with delirium. A stepwise selection process with entry criteria of *P* = .05 was used to select variables that were independently associated with delirium. The independent predicting ability of predictors was expressed by the odds ratio (OR) value and the 95% confidence interval (95% CI). The area under the receiver operating characteristics curve (AUROC) were subsequently used to evaluate risk factors for predicting the likelihood of pediatric patients to develop delirium. All statistical tests were 2-sided with statistical significance evaluated at the 0.05 alpha level. All analyses were performed in SPSS, version 26.0 (IBM Corporations, Armonk, NY).

## 3 Results

### 3.1 Characteristics of patient population

One thousand one hundred thirty-four subjects were enrolled and 6691 patient days were evaluated. Each patient was assessed for delirium after surgery. Demographic and clinical patient information is presented in Table 1. These patients ranged in age from 1day to 16 years, about 60% of patients were younger than 5 years old, 60% were male, 4% were developmentally delayed (developmental delay was defined as sever impairment in ability to communicate in age-appropriate way with caregiver at pre-hospital baseline), and 82% had American Society of Anesthesiologists grade I. Nearly 32% patients had otorhinolaryngology surgery, 27% had orthopedic surgery, and 27% had thoracic and abdominal surgery. During the assessments, about 97% were prescribed propofols, 86% benzodiazepines, 60% corticosteroids, 51% anticholinergics, 58% dexmedetomidines, 58% opioid receptor agonists, 51% neuromuscular blockers.

**Table 1:** Univariate analysis of clinical patient characteristics (N = 1134).

Characteristic	Total	Delirium	No Delirium	*P* value
N	1134	126 (11.11%)	1008 (88.89%)	–
Gender				.129^∗^
Male	676 (59.61%)	83 (65.87%)	593 (58.83%)	
Female	458 (40.39%)	43 (34.13%)	415 (41.17%)	
Age, yr				<.001^∗^
0–2	368 (32.45%)	76 (60.32%)	292 (28.97%)	
>2–5	316 (27.87%)	42 (33.33%)	274 (27.18%)	
>5–16	450 (39.68%)	8 (6.35%)	442 (43.85%)	
Developmental Delay				<.001^∗^
No Delay	1089 (96.03%)	113 (89.68%)	976 (96.83%)	
Delay	45 (3.97%)	13 (10.32%)	32 (3.17%)	
Pre-existing medical conditions				.006^∗^
No	1054 (92.95%)	125 (99.21%)	929 (92.16%)	
Yes	80 (7.05%)	1 (0.79%)	79 (7.84%)	
ASA classification				.233^∗^
I	926 (81.66%)	98 (77.78%)	828 (82.14%)	
II	208 (18.34%)	28 (22.22%)	180 (17.86%)	
Type of Surgery				<.001^∗^
Otorhinolaryngology surgery	363 (32.01%)	59 (46.82%)	304 (30.16%)	
Thoracic and abdominal surgery	305 (26.90%)	21 (16.67%)	284 (28.17%)	
Orthopedic surgery	309 (27.25%)	25 (19.84%)	284 (28.17%)	
Other surgery	157 (13.84%)	21 (16.67%)	136 (13.49%)	
Sleep duration (h), median (IQR)	8.78 (8.00,9.50)	8.58 (8.00,9.50)	8.75 (8.00,9.50)	.687^#^
Fluid fasting time (h), median (IQR)	12.00 (9.00,14.86)	11.31 (8.96,13.12)	12.21 (9.00,15.00)	.002^#^
Parental Anxiety Score				.003^∗^
<7	773 (68.17%)	71 (56.35%)	702 (69.64%)	
≥7	361 (31.83%)	55 (43.65%)	306 (30.36%)	
Length of anesthesia (min), median (IQR)	58.00 (43.00,78.50)	52.00 (40.00,81.00)	58.00 (45.00,78.00)	.040^#^
Length of surgery (min), median (IQR)	30.00 (20.00,50.00)	28.00 (18.75,56.00)	30.00 (20.00,50.00)	.108^#^
Blood Loss (ml), median (IQR)	2.00 (1.00,5.00)	1.00 (1.00,5.00)	2.00 (1.00,5.00)	.153^#^
Pain				<.001^∗^
Mild or painless (0-3)	980 (86.42%)	43 (34.13%)	937 (92.96%)	
Moderate (4–6)	98 (8.64%)	45 (35.71%)	53 (5.26%)	
Severe (7–10)	56 (4.94%)	38 (30.16%)	18 (1.78%)	
Fever				.203^∗^
No	925 (81.57%)	108 (85.71%)	817 (81.05%)	
Yes	209 (18.43%)	18 (14.29%)	191 (18.95%)	
Medication exposures				
propofol				.832^∗^
No	35 (3.09%)	3 (2.38%)	32 (3.17%)	
Yes	1099 (96.91%)	123 (97.62%)	976 (96.83%)	
Benzodiazepine				.928^∗^
No	159 (14.02%)	18 (14.29%)	141 (13.99%)	
Yes	975 (85.98%)	108 (85.71%)	867 (86.01%)	
Corticosteroids				.915^∗^
No	455 (40.12%)	50 (39.68%)	405 (40.18%)	
Yes	679 (59.88%)	76 (60.32%)	603 (59.82%)	
Anticholinergics				.053^∗^
No	560 (49.38%)	52 (41.27%)	508 (50.40%)	
Yes	574 (50.62%)	74 (58.73%)	500 (49.60%)	
Dexmedetomidine				.027^∗^
No	473 (41.71%)	41 (32.54%)	432 (42.86%)	
Yes	661 (58.29%)	85 (67.46%)	576 (57.14%)	
Opioid Receptor Agonist				.718^∗^
No	476 (41.98%)	51 (40.48%)	425 (42.16%)	
Yes	658 (58.02%)	75 (59.52%)	583 (57.84%)	
Neuromuscular blocker				.628^∗^
No	554 (48.85%)	59 (46.83%)	495 (49.11%)	
Yes	580 (51.15%)	67 (53.17%)	513 (50.89%)	
Ibuprofen suppositories				.734^∗^
No	1078 (95.06%)	119 (94.44%)	959 (95.14%)	
Yes	56 (4.94%)	7 (5.56%)	49 (4.86%)	

### 3.2 Delirium incidence

**Figure 2:**
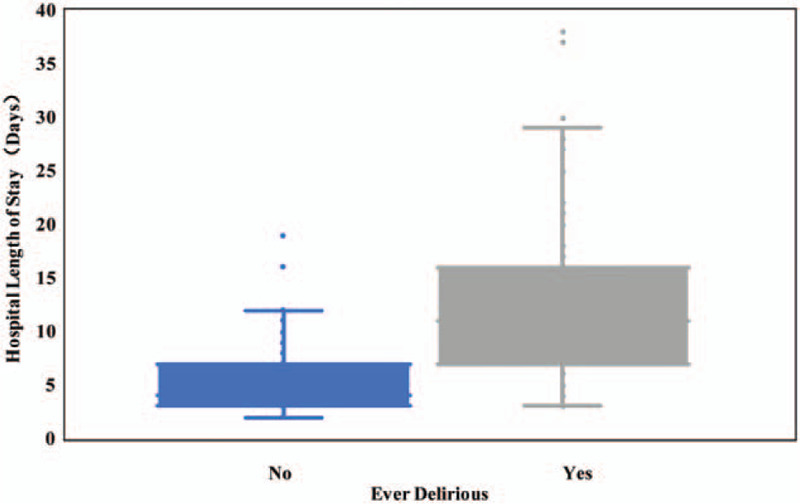
The left box plot shows the hospital length of stay for patients who were never diagnosed with delirium. The right box plot shows the hospital length of stay for patients who were delirious. The hospital length of stay estimates shows the significative difference between delirium group and no delirium group.

One hundred twenty-six patients were diagnosed with delirium, and the prevalence of delirium was 11.1%. Delirium most often developed within the first 15 to 145 minutes (median 40, IQR 35–50) after surgery. Duration of delirium ranged from 1 to 60 minutes, with an IQR of 5 to 15 minutes, and a median of 10 minutes. Median hospital LOS was 4 days, with an IQR of 3 to 7days. LOS for patients diagnosed with delirium was significantly longer than LOS for patients who were never diagnosed with delirium (median=4 days vs 11days; *P* < .001) (Fig. 2).

### 3.3 Factors associated with the onset of delirium

In univariate analyses, pre-existing factors associated with diagnosis of delirium included age, developmental delay, pre-existing medical conditions, and the type of surgery. Surgery related factors linked to fluid fasting time, parental anxiety, length of anesthesia, pain score, and receipt of dexmedetomidine (Table 1).

In multivariable modeling, adjusted ORs showed an independent association between development of delirium and age, developmental delay, type of surgery, pain, and administration of dexmedetomidine (Table 2). The adjusted odds for delirium diagnosis were almost 10 times greater in patients who were infants (0–2 years old), as compared with school age [>5–16 years old, OR = 9.944 (4.361, 22.676); *P* < .001]. Presence of developmental delay [OR = 4.070 (1.584, 10.454); *P* = .004] was strongly associated with delirium. Otorhinolaryngology surgery were associated with postoperative delirium compared to orthopedic surgery, thoracic and abdominal surgery (*P* < 0.05). Moderate pain [OR = 17.032 (9.603, 30.210); *P* < .001] and severe pain (OR=42.717(20.443, 89.259); *P* < .001) were found to be statistically significant in predicting pediatric delirium when compared with mild or painless. Exposure to dexmedetomidine [OR=1.947 (1.160, 3.270); *P* = .012] were strongly associated with pediatric delirium. After step-wise selection, parental anxiety score, length of anesthesia, pre-existing medical conditions, and type of surgery fell out of the final model.

**Table 2:** Multivariable logistic regression analysis predicting delirium (N = 1134).

Variable	β	S.E.	Wald	*P*	OR	95%CI
Constants	−4.688	0.466	101.338	<.001	0.009	–
Age, yr	–	–	30.021	<.001	–	–
>2-5	1.773	0.427	17.225	<.001	5.888	2.549–13.602
0-2	2.297	0.421	29.828	<.001	9.944	4.361–22.676
Developmental Delay	1.404	0.481	8.504	.004	4.070	1.584–10.454
Type of Surgery	–	–	13.790	.003	–	–
Orthopedic Surgery	−1.217	0.343	12.608	<.001	0.296	0.151–0.580
Thoracic and Abdominal Surgery	−0.677	0.324	4.359	.037	0.508	0.268–0.959
Other Surgery	−0.482	0.362	1.779	.182	0.617	0.304–1.254
Pain	–	–	145.902	<.001	–	–
Moderate	2.835	0.292	94.020	<.001	17.032	9.603–30.210
Severe	3.755	0.376	99.713	<.001	42.717	20.443–89.259
Dexmedetomidine	0.666	0.264	6.353	.012	1.947	1.160–3.270

### 3.4 Development of prediction model

**Figure 3:**
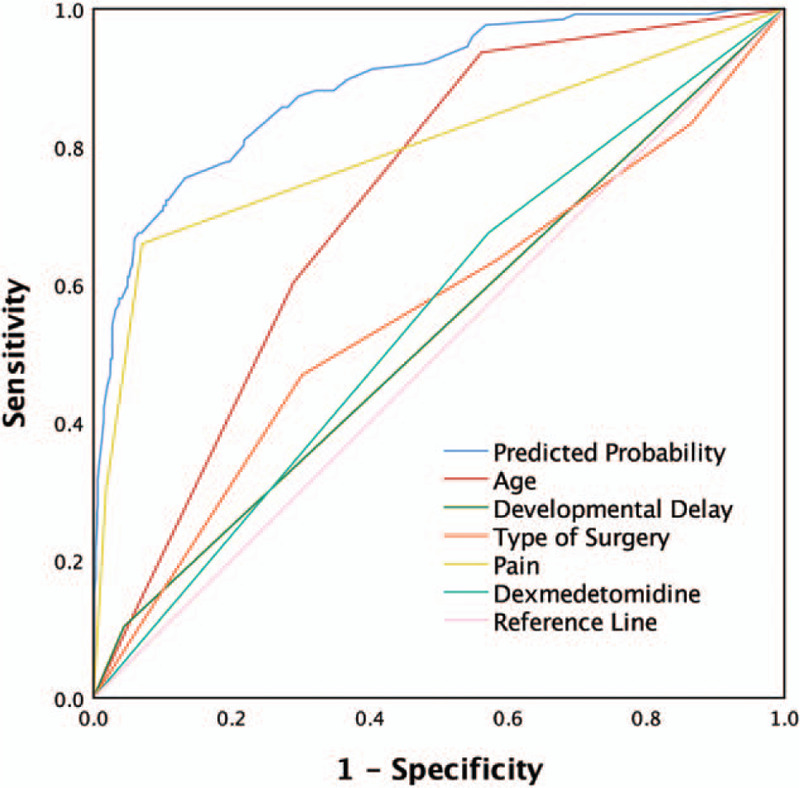
The area under the receiver operating characteristics curve (AUROC) of predicted probability shows that the predictive ability of the entire model is better than a single predictive factor.

**Figure 4:**
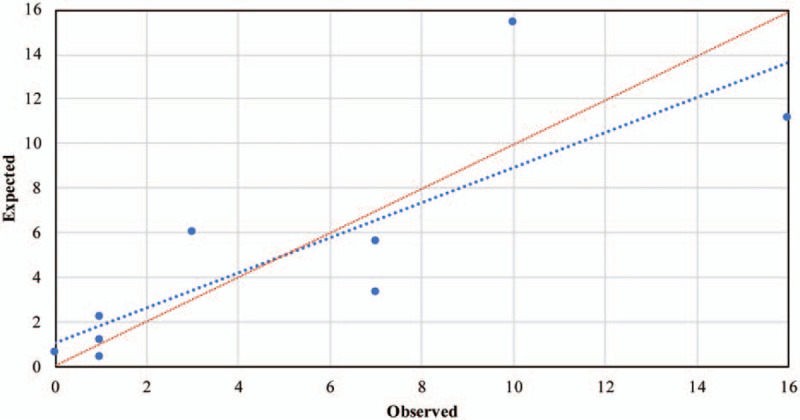
The blue line is the calibration curve, and the red line is the standard curve. The closeness of the calibration curve and the standard curve shows the calibration capability of the model.

Using Logit(P/1-P) as the dependent variable and assigning independent variables (Table 3), a logistic regression model containing 5 independent variables was established: Logit (P/1-P) = − 4.688 + 1.733 × X_1_B + 2.297 × X_1_C + 1.404 × X_4_ − 1.217 × X_2_B − 0.677 × X_2_C − 0.482 × X_2_D + 2.835 × X_3_B + 3.755 × X_3_C + 0.666 × X_5_. The likelihood ratio test of the logistic regression model shows that the global test of the model is statistically significant (*χ*^*2*^ = 305.908, *P* < .001). The Hosmer-Lemeshow goodness-of-fit test shows that the model fits well (*χ*^*2*^ = 12.833, *P* = .118). The model prediction accuracy rate is 92.1%, indicating that the model prediction effect is relatively ideal. The results show that the predictive power of the entire model is better than a single predictive factor (Table 4). The AUROC is 0.889 (*P* < .001, 95%CI: 0.857–0.921), the maximum Youden index is 0.621, the sensitivity is 0.754, and the specificity is 0.867, which shows that the model has high predictive performance (Fig. 3). According to the model prediction formula, when *P* ≥ .621, it is considered that the patients will develop delirium after surgery. The model calibration capability is shown in Figure 4.

**Table 3:** Variable assignment.

Code	Variables	Assignment
X_1_	Age	>5–16 = A, >2–5 = B, 0–2 = C
X_2_	Type of Surgery	Otorhinolaryngology Surgery = A, Orthopedic Surgery = B, Thoracic and Abdominal Surgery = C, Other Surgery = D
X_3_	Pain	Mild or painless = A, Moderate = B, Severe = C
X_4_	Developmental Delay	–
X_5_	Dexmedetomidine	–
X_6_	Pre-existing medical conditions	–
X_7_	Parental anxiety	–

**Table 4:** Area under receiver operating characteristics curve for different factors of postoperative delirium prediction model in pediatric patients.

Variable	AUROC	S.E.	*P*	95%CI
Model	0.889	0.016	<.001	0.857–0.921
Age	0.721	0.020	<.001	0.681–0.761
Developmental Delay	0.536	0.029	.191	0.480–0.592
Type of Surgery	0.557	0.030	.038	0.499–0.614
Pain	0.799	0.026	<.001	0.748–0.850
Dexmedetomidine	0.552	0.027	.059	0.499–0.604

### 3.5 Validation of the prediction model

We further collected data from 100 patients in October 2020 to validate the model. The newly created model was used to predict the probability of delirium in 100 patients. The AUROC of the model was 0.919 (*P* < .001, 95%CI:0.853–0.985). The sensitivity of the model was 0.667, and the specify was 0.955.

## 4 Discussion

Postoperative delirium is a serious complication that relates to an increase in length of hospital stay and poor outcomes.^[22]^ It is noteworthy that despite numerous studies focused on pediatric delirium, the prevalence and risk factors of postoperative pediatric delirium are still not clear in China. In this study, we focused on surgical patients in order to attract people's attention to children undergoing major elective surgery and to find a strategy to prevent postoperative delirium.

Based on data from over 1000 patients, we found that nearly 1 in 10 children developed delirium after surgery. Without routine screening, these children may be underdiagnosed. Therefore, screening for postoperative delirium should be initiated at once after surgery. Over the past few years, the reported prevalence of pediatric delirium ranging from 4.5% to 65.9%,^[19,23]^ our findings are considerably lower than the 10% to 30% delirium rates reported in the general population of critically-ill children.^[24]^ A prospective single-center study found that prevalence of delirium was 49% in children after cardiac bypass surgery.^[23]^ Another single-center prospective cohort study found that nearly 66% children were delirious after major surgery in pediatric intensive care unit (PICU).^[19]^ This may be explained by following aspects. First, we focused on all children after major elective surgery rather than critically-ill children in PICU. Second, some short courses of postoperative delirium might have been missed. Third, the risk of developing delirium seems to be closely related to young age. In our cohort, only 32% of the patients were younger than 2 years old compared to about half the patients under 2 years old in other studies.

Although the pathophysiology of pediatric postoperative delirium is still incompletely understood, it is likely a syndrome resulting from a multifactorial process that involves preoperative, perioperative, and postoperative factors. Consistent with the risk factors described in the PICU, delirium in our cohort was associated with baseline and disease related risk factors.^[25]^ Several recent studies of pediatric delirium demonstrated an association between delirium and extremely young age, severity of illness, need for mechanical ventilation, and pharmacologic sedation.^[23,26]^ In our study, we found that young age (<5yr), developmental delay, severity of pain, and exposure to anticholinergics and dexmedetomidine were independently associated with increased risk of postoperative pediatric delirium.

There are nonmodifiable demographic risk factors associated with postoperative pediatric delirium, such as young age and developmental delay. In our study, 60.32% of the patients were younger than 5 years old, and the odds of delirium were 5.89 times higher for patients older than 2 to 5 years old, 9.94 times higher in patients under than 2 years old. Owing to the development of the cholinergic function and hippocampus in the first 3 years of developing brain, children might at a greater risk for pediatric delirium during this period.^[27]^ The AUROC of age is 0.721, which indicating that age is effective in predicting postoperative delirium in children. This suggests that medical staff should pay attention to younger children after surgery. Consistent with previous literature, this study suggests that preoperative patient vulnerability is a risk factor, as children with developmental delay were at higher risk for delirium. In this study, 10% children who ever delirious are developmental delay, and the odds of delirium were 4 times higher for this population. An atypical brain at baseline may be more vulnerable to the effect of surgery, as well as adults with dementia were at high-risk of developing delirium in adult patients. The AUROC of developmental delay was 0.536 (*P* = .191), indicating that developmental delay alone has no predictive effect on postoperative delirium in children.

The risk factors for delirium in our cohort support previous work within the field. Literatures in adult postoperative delirium researches have shown a strong association between development of delirium and surgery related factors, such as type of surgery and pain.^[28]^ Among children with postoperative delirium in our study, 46.82% had otorhinolaryngology surgery, 19.84% had orthopedic surgery, and 16.67% had thoracic and abdominal surgery. Concerning pain, the odds of delirium were 17 times higher for patients with moderate pain, and 42 times higher for patients with severe pain compared to painless patients. It is interesting to note that 34% patients report mild pain or painless after recovering normal state, which supports that postoperative delirium also occurs following nonpainful procedures. The AUROC of pain was 0.799, indicating that pain is effective in predicting postoperative delirium in children. This suggests that medical staff should promptly assess the degree of postoperative pain in children. Drug and nondrug intervention measures should be applied to relieve postoperative pain and prevent the occurrence of delirium.

Finally, consistent with both adult and pediatric research in delirium, we demonstrated an association with delirium development and dexmedetomidine.^[29]^ In this study, we observed increased odds of delirium with dexmedetomidine exposure compared with no exposure. The AUROC of dexmedetomidine was 0.552 (*P* = .059), which confirms that postoperative delirium is a result of multifactorial effect. Although dexmedetomidine has been associated with lower risk of delirium in adult randomized controlled trials compared with other sedatives, it is not associated with zero risk.^[30–31]^ It raised concerns that sedatives may not be risk-free.

## 5 Limitations

This study has several limitations. For the most part, our study was performed in a single center, the prevalence of delirium reported here may not be widely generalizable. Further multicenter studies are necessary to confirm these findings. Secondly, it is possible that children may not have demonstrated the fluctuating symptoms of delirium during the assessment time, and children who were delirious at night may be missed, which may have falsely lowered the delirium prevalence measured in our study. Thirdly, although our data collection included many possible risk factors for delirium development, identified from the adult postoperative delirium literature, it is possible that other unknown and important risk factors were missed. In addition, we did not capture delirium subtype (hypoactive, hyperactive, or mixed), this is an important area of focus for future studies. Lastly, we did not collect data regarding particular medication used, or doses, which are recommended to be included into the model in subsequent studies.

## 6 Conclusion

We successfully developed a multivariate logistic regression equation by using 5 related factors to predict postoperative delirium in pediatric patients. This model shows the relationship between delirium-related risk factors and delirium intuitively in the formula, which helps health care professionals pay more attention to the potential risk of postoperative delirium in pediatric patients. We find that children undergoing surgery are at risk for developing delirium during the postoperative period. The risk factors of postoperative delirium include young age, developmental delay, type of surgery, pain, and exposure to dexmedetomidine. The findings from our study remind us that it is important to screen delirium after surgery, and several in-hospital risk factors for delirium development are modifiable. Interventional studies are warranted to assess treatment and prevention strategies in postoperative pediatric delirium.

## Acknowledgments

The authors of this paper wish to acknowledge the contributions of nurses who contributed to data collection. We also wish to acknowledge all the children who participated in this study.

## Author contributions

**Conceptualization:** Nan Lin, Kexian Liu, Hongzhen Xu.

**Data curation:** Nan Lin, Jingyi Feng, Ruan Chen, Yan Ying, Danni Lv, Yue Zhou.

**Funding acquisition:** Hongzhen Xu.

**Methodology:** Nan Lin.

**Resources:** Hongzhen Xu.

**Software:** Nan Lin.

**Validation:** Kexian Liu.

**Writing – original draft:** Nan Lin.

**Writing – review & editing:** Nan Lin.
